# The Short-Term Effects of European Integration on Mortality Convergence: A Case Study of European Union’s 2004 Enlargement

**DOI:** 10.1007/s10680-021-09596-y

**Published:** 2021-10-07

**Authors:** Rok Hrzic, Tobias Vogt, Helmut Brand, Fanny Janssen

**Affiliations:** 1 Department of International Health, Care and Public Health Research Institute – CAPHRI, Maastricht University, Maastricht, The Netherlands; 2 International Max Planck Research School for Population, Health and Data Science, Max Planck Institute for Demographic Research, Rostock, Germany; 3 Faculty of Spatial Sciences, Population Research Centre, University of Groningen, Groningen, The Netherlands; 4 Prasanna School of Public Health, Manipal Academy of Higher Education, Manipal, India; 5 Max Planck Institute for Demographic Research, Rostock, Germany; 6 Netherlands Interdisciplinary Demographic Institute – KNAW / University of Groningen, The Hague, The Netherlands

**Keywords:** Convergence, Economic integration, Europeanisation, Political determinants of health, Regional studies, Transition

## Abstract

Although European integration can be expected to result in mortality convergence (reduced mortality differences), a life expectancy divide persists in the European Union (EU) between the old Member States (OMS) in the west and the new Member States (NMS) in the east. Studies investigating the impact of European integration on mortality convergence are rare and did not consider regional differences. We examine the short-term effects of the 2004 enlargement on mortality convergence at the supranational, national, and subnational levels. Using sex-specific life expectancies for 23 Member States (1990–2017) and the NUTS 2 regions in Czechia, Hungary, and Poland for 1992–2016, we examined the trend in sigma and beta mortality convergence measures at the country and regional levels using joinpoint regression. We found no compelling evidence that EU accession influenced the process of mortality convergence between OMS and NMS, or within the three NMS, over the short term. While there was overall beta and sigma convergence at the national level during 1990–2017, no regional convergence showed, and the trends in convergence did not significantly change at the time of EU accession or soon after (2004–2007). The accession in 2004 did not visibly impact the overall process of mortality convergence over the short term, likely because of the greater influence of country and region-specific policies and characteristics. The interaction of Member State and regional contexts with the mechanisms of European integration requires further study. Future enlargement procedures should emphasise tailored support to ensure more equitable gains from European integration.

**Supplementary Information:**

The online version contains supplementary material available at 10.1007/s10680-021-09596-y.

## Introduction

There is a historical east–west European gap in life expectancy, with life expectancy in the eastern part of the continent—i.e. in the countries that were formerly behind the Iron Curtain—lagging behind life expectancy in the west for the past half century (Bobak & Marmot, 1996; Leon, 2011; Meslé & Vallin, 2002). At the time of the first eastward enlargement of the European Union (EU) in 2004, the average gap between the new Member States in the east and the old Member States in the west was seven years for men and four years for women (Eurostat, 2020a). The 2004 enlargement made the east–west life expectancy gap a central feature of geographic disparities in health in the EU, and became a source of concern for EU policy-makers.

One of the aims of the European integration process is to bring about economic and social convergence between the accession countries and the established member states (European Commission, 2020), including convergence (reduced differences over time) in health and mortality. Therefore, it is important to understand the effects of European integration on mortality convergence not just from an academic perspective, but also from a societal perspective. Moreover, given that a further eastward expansion of the EU is planned, examining this issue can contribute to the ongoing discussion on revising the accession procedures (European Commission, 2020). In particular, our paper sheds light on whether the current accession procedure is a strong determinant of rapid mortality convergence, thus clarifying what current EU candidate countries in the Western Balkans may reasonably expect from accession in terms of mortality outcomes over the short term, and indicates how they can best seize the opportunities of European integration to improve population health.

The conditions under which mortality convergence—i.e. the mortality or life expectancy trajectories of populations becoming increasingly similar over time—might occur have been previously theorised by Vallin and Meslé (Meslé & Vallin, 2017; Vallin & Meslé, 2004, 2005). In brief, they argued that mortality divergence is a consequence of countries adopting medical technologies, public health policies, and health behaviours at different speeds. Their theory splits countries into the vanguard group, which consists of the countries that developed and implemented mortality-reducing innovations first; and the laggard group, which consists of the countries that are catching up to the vanguard at varying speeds, depending on the general rate of diffusion of the innovations and the laggards’ ability to absorb the innovations. They emphasised that the process of divergence and convergence follows complex divergence–convergence cycles, several of which may occur simultaneously (see next section for additional detail).

We believe European integration could influence mortality convergence over the short term because political and economic integration had previously contributed to rapid mortality convergence. The key example of this is post-reunification Germany. During the 1980s, life expectancy in East Germany lagged behind West Germany by 6 years in men and 4 years in women, and this gap was closed for women and reduced to 1 year within a decade of reunification (Grigoriev & Pechholdova, 2017). To what extent the mortality convergence observed there is due to the reunification policies compared to other changes is a subject of ongoing research. However, previous work shows that the reunification likely sped up existing trends towards mortality convergence (Grigoriev & Pechholdova, 2017) and that reunification-associated policies like increased social welfare spending and hospital infrastructure investments in former East Germany likely played a significant role (Vogt, 2013; Vogt & Kluge, 2015; Vogt & Vaupel, 2015).

We also believe European integration may contribute to rapid mortality convergence due to the mechanisms of EU accession. Here we highlight two possible pathways between accession and mortality convergence. The first acts through economic integration and access to EU funding (e.g. cohesion fund), which speeds up economic development (Bachtler, 2013) and the capacity to absorb new innovations in the new Member States, and thus improves their mortality conditions (Mackenbach & Looman, 2013; Preston, 1975). The second acts through improved governance, which generally entails stabilising institutions; integrating the body of EU law into national legislation [also known as the “hard” *acquis communautaire* (Gialdino, 1995)]; and adopting best practices through benchmarking, access to EU expertise, and the open method of coordination [part of the “soft” *acquis* (cf., Trubek & Trubek, 2005)]. Stable and similar institutions and legal environments tend to increase the rate of diffusion of innovations between the new and the old Member States. This can lead to convergence in social and health policies and in the quality of health care, which, in turn, should improve mortality conditions in new Member States (Albreht, 2014; Ostojic et al., 2012).

Studies have shown that European integration influenced economic convergence, but with important differences between countries and regions. For example, studies that examined the effects of the 2004 enlargement generally found that convergence in per capita gross domestic product between the new and the old Member States was underway since the enlargement was announced in the mid-1990s, but that the convergence was slowed in the aftermath of the Great Recession (Dobrinsky & Havlik, 2014). The literature also documents considerable differences in economic growth between the new Member States as well as between regions within Member States, causing some regions to persistently lag behind (Cutrini, 2019; Dobrinsky & Havlik, 2014; Iammarino et al., 2019).

While there is a rich literature on mortality convergence in Europe (see next section for a brief summary), studies that explicitly investigated the impact of European integration on mortality convergence are rare. Mackenbach (2013) touched on the issue in the context of the enlargements of the 1980s and 1990s, and found no signs of convergence between the six founding members and the accession countries following their entry into the EU. Santos and colleagues (2020) aimed to study the immediate impact of enlargements on the average EU health status, and found that the 2004, 2007, and 2013 enlargements resulted in a significant worsening of the EU’s average standardised mortality rate, life expectancy, and healthy life expectancy.

However, these studies have two important limitations. First, they did not probe the potential heterogeneity of the effects of European integration—as observed for economic convergence—and its impact on the overall process of mortality convergence. Second, they either did not include the eastern enlargements in their analysis, or they addressed several EU enlargements at once, even though each enlargement process involved a unique set of accession procedures and countries (cf., Bachtler, 2013; Hille & Knill, 2006).

We seek to address this gap by analysing the short-term effects of European integration and EU membership in particular on mortality convergence at three geographic levels: the supranational, national, and subnational levels.

We consider the 2004 enlargement a particularly interesting context for studying the effects of European integration on life expectancy convergence for the following reasons: (1) as the enlargement was the largest ever in terms of population size and number of accession countries, it provides a sizeable sample to observe; (2) because the gap in life expectancy between the new and the old Member States was substantial, there was ample scope for convergence; (3) sufficient time has passed to allow a balanced time series of regional-level data from before and after accession to emerge; and (4) the enlargement was the first to use the accession procedure reformed by the introduction of the Copenhagen and Madrid criteria in 1993 and 1995 (Vasileiou, 2007), which still apply.

## The Origin and Development of the Present East–West Life Expectancy Gap in Europe

The divergence-convergence cycle that resulted in the present east–west life expectancy gap in Europe emerged in the late 1960s and the early 1970s (Bobak & Marmot, 1996; Leon, 2011). While mortality due to cardiovascular disease and external causes (alcohol-related deaths and accidents) remained stagnant in central Europe and rose in eastern Europe, other European countries experienced substantial decreases (Meslé & Vallin, 2002). The most likely drivers of this divergence were the cardiovascular revolution and the successful prevention of other “man-made” diseases in Western European countries (Meslé & Vallin, 2002).

The term cardiovascular revolution encompasses healthcare interventions that reduced mortality after the onset of cardiovascular disease, including advances in pharmacology, cardiovascular surgery, and improved emergency services, as well as the declining prevalence of harmful health-related behaviours (poor diet, physical inactivity, tobacco and alcohol consumption) that reduced the incidence of cardiovascular disease (Grigoriev et al., 2014; Vallin & Meslé, 2004). These changes significantly reduced cardiovascular mortality in the west, but their adoption and resulting mortality benefit were delayed in countries of central and eastern Europe. The likely causes of the delay were the relative isolation from the west, economic difficulties, and the overly centralised care provision, all of which made the diffusion of the innovations in medical technology and health policies more difficult (Meslé & Vallin, 2002, 2017).

Another contributor to the present east–west gap was the mortality crisis in post-Soviet Russia and the Baltic states, which entailed a sharp drop in life expectancy in the 1990s, followed by a rapid recovery for the Baltics (Jasilionis et al., 2011). The causes of the mortality crisis were traced to abandoning of Gorbachev era anti-alcohol policies combined with the socio-economic shock of the rapid economic transition, which resulted in a rapid increase in deaths due to suicide, injury, and alcohol-related deaths (Grigoriev et al., 2010).

A rich literature, including a recent special issue of the European Journal of Population (Meslé & Vallin, 2017), describes the country-specific patterns and drivers of the mortality convergence as part of the divergence-convergence cycle in post-2004 EU Member States. This includes studies that examined Czechia and Poland (Fihel & Pechholdová, 2017; Nolte, 2000; Nolte et al., 2000, 2002; Rychtarikova, 2004), where rapid mortality improvements began in the early 1990s, and the Baltic states (Grigoriev et al., 2010; Jasilionis et al., 2011; Lai & Habicht, 2011; Stumbrys et al., 2020), where Estonia emerged as the leader in life expectancy gains in the 2000s. The authors show that a combination of several factors drives divergence and convergence in mortality, including accessibility and quality of healthcare, health system reforms, social welfare and health policies—particularly alcohol policy—and changes in health-related behaviours.

Studies have also examined mortality trends in geographic Europe more generally (Gerry et al., 2018; Timonin et al., 2016), and mortality convergence at the regional level in the enlarged EU (Jaworska, 2014; Maynou et al., 2015). These studies highlight the heterogeneity in life expectancy trends between countries and regions, and show that they tend to coalesce into distinct groups, also called convergence clubs.

This body of literature provides incisive insights into the country-specific drivers of mortality convergence. However, while past international developments, for example, the dissolution of the Soviet Union, have been featured in the literature as impetus for domestic policy change, current supranational developments in Europe, most notably the enlargement of the European Union, have been less often studied as potential drivers of convergence.

## Methods

### Study Design

To study the short-term effects of the 2004 EU enlargement on mortality convergence at the supranational, national, and subnational levels, we analysed short-term shocks to convergence in period life expectancy at birth (henceforth LE) in the enlarged EU during 2004–2007, separately for men and women. We have chosen this time window instead of a longer one to be able to distinguish between the effects of EU membership, and the effects of two other supranational events that may have impacted life expectancy convergence, i.e. the Great Recession (2007–2009), the European sovereign debt crisis (2009–2014), and their associated policy responses (Stuckler et al., 2017).

We situate the 2004–2007 time window within the wider study period 1990–2017, thereby distinguishing: (1) the pre-accession period (1990–2003), when social and economic reforms in the future NMS were undertaken and the association agreements (“Europe Agreements”) that liberalised trade and investment between future NMS and OMS took effect (Vasileiou, 2007); and (2) the EU membership period (2004–2017), when the direct benefits of the full membership in the single market and the EU governance system may be observable.

We focus on the 23 continental post-2004 EU Member States, excluding Malta and Cyprus, and divide them into the new Member States group (NMS: Czechia, Estonia, Hungary, Latvia, Lithuania, Poland, Slovenia, and Slovakia) and the old Member States group (OMS: Austria, Belgium, Denmark, France, Germany, Greece, Finland, Ireland, Italy, Luxembourg, the Netherlands, Portugal, Spain, Sweden, and the United Kingdom).

Our study design involves examining the convergence between the new Member States group and old the Member States group (supranational level), mortality convergence within each of the country groups (national level), as well as regional mortality convergence within Member States (subnational level). For the regional-level analysis, we focused on NUTS 2 regions, which correspond to existing administrative units or to ad hoc geographical, socio-economic, historical, cultural or environmental aggregations with population sizes that are generally between 800,000 and three million. Moreover, NUTS 2 regions are the basic regions for the application of regional policies, including the EU’s cohesion policy (European Commission, 2019). We limited the regional-level analysis to Czechia, Hungary, and Poland during 1992–2016 due to poor regional data availability for the other new Member States prior to EU accession, as well as gaps in regional data in the most relevant old Member States, especially Germany. The data made publicly available by the national or state statistics offices did not fully address the missing data, or we had concerns about the comparability of these data. See Supplementary Table 1 for an overview of regional data availability.

### Data

We extracted sex-specific LE and population counts for the 23 Member States for the period 1990–2017 from the Human Mortality Database (2021). We also extracted sex-specific LE for NUTS 2 regions in Czechia, Hungary, and Poland for the period 1992–2016 from Eurostat (Eurostat 2020a).

The Czech regional data, comprising 8 NUTS 2 regions, were complete for 1992–2016 at time of extraction. The Hungarian and Polish regional LE data had significant gaps owing to changes in the NUTS classification. We therefore used data for these countries that conformed to the older NUTS 2013 classification, which included 7 NUTS 2 regions for Hungary and 16 NUTS 2 regions for Poland, and which were still available from Eurostat in late 2019. We also used linear interpolation to fill in single missing years of data for regions in Poland (year 2001 for all regions), where the LE for the missing year for each region was estimated as the average of the LE in the preceding and following years.

### Measures of Mortality Convergence

We assessed mortality convergence from two perspectives. The first perspective is unconditional beta convergence in LE. We use the standard definition of unconditional beta convergence (Sala-i-Martin, 1996), which has been used in previous analyses of mortality convergence (Gächter & Theurl, 2011; Janssen et al., 2016; Timonin et al., 2017), where unconditional beta convergence stands for an inverse association between starting LE and the change in LE during a defined period.

As beta convergence is a necessary but insufficient condition of convergence, we complement it with the second perspective, sigma convergence. We operationalised sigma convergence as a reduction in dispersion in LE over time (Sala-i-Martin, 1996). Since dispersion measures differ in their mathematical properties and ability to summarise a distribution (Cowell, 2011), it is advantageous to report several to provide a comprehensive picture of convergence. We measured LE dispersion using both the variance and the Theil index (Theil, 1967). Both measures are additively decomposable and have been previously used in convergence studies in the EU context (see Hrzic et al., 2020 for a review). The Theil index is also robust to changes in the mean of the distribution (Cowell, 2011). The measures differ in that variance uses the absolute difference as the measure of distance while the Theil index uses a proportional measure of distance.

### Statistical Analysis

#### Beta Convergence

We assessed beta convergence by examining—by linear regression—the association between LE of country or region i at the start of a period (${\text{LE}}_{{i,t_{1} }}$) and the annual change in LE in country or region i between *t*_1_ and *t*_2_:

$$\Delta {\text{LE}}_{{i,t_{2} - t_{1} }} = \alpha + \beta {\text{LE}}_{{i,t_{1} }} + \varepsilon$$

If the relationship is inverse ($\beta$ is negative) and if the association is statistically significant, we conclude that beta convergence occurred between *t*_1_ and *t*_2_. Both unweighted and population-weighted regressions were performed, the latter using weighted least squares for estimation.

We performed this analysis for the full period 1990–2017 (1992–2016 for the regional-level analysis), as well as for overlapping four-year periods (1990–1994, 1991–1995, …) and for overlapping six-year periods (1990–1996, 1991–1997, …) and thereby constructed a time series of beta coefficients. We then examined this time series for significant changes during 2004–2007, our time window of interest.

#### Sigma Convergence

To assess life expectancy dispersion, we calculated the variance and Theil index of LE for each sex and year included in the analysis. If dispersion was smaller at *t*_2_ compared to *t*_1_, we concluded that sigma convergence occurred in the period between *t*_1_ and *t*_2_. Confidence intervals for the value of the dispersion measures at each point in time and their differences over time were calculated via the nonparametric bootstrap approach (Mills and Zandvakili, 1997).

We used both population-weighted and equally-weighted variants of both dispersion measures. Whereas the population-weighted measures reflect both disparities between places as well as their relative population sizes, the unweighted measures focus on disparities between places (Gluschenko, 2018). The population-weighted measures therefore emphasise the experience of Member States and regions with larger populations at the expense of less populous ones. Since our study aims to reflect heterogeneity in convergence and therefore seeks to highlight the experience of less populous Member States and regions, we added equally-weighted variants as well.

We decomposed the total LE dispersion into the dispersion between the OMS and the NMS country groups and the dispersion between the countries within the OMS and the NMS country groups (Conceição & Ferreira, 2000), using established one-stage decomposition procedures (Akita, 2003; Shorrocks & Wan, 2005).

#### Analyis of Trends

To examine trend changes in beta and sigma convergence measures, and how these trend changes correspond to EU accession, we used linear joinpoint regression (Kim et al., 2000). There are two main approaches to joinpoint regression. In the first approach, the researchers hypothesise the number and location of joinpoints (i.e. breaks in the trend line) and fit the corresponding model (Bernal et al., 2017). The second approach, which we use in this study, is data driven. In this approach, the location of joinpoints is not selected a priori*,* but is instead empirically determined by fitting models assuming different numbers and locations of joinpoints, and selecting the model that best fits the data (Kim et al., 2000). The interpretation in this approach relies on the empirically determined locations of the joinpoints and their fit with the timing of the event, in our case the time window 2004–2007. The data-driven approach is more appropriate for studies in which the lag between the onset of the intervention and its effects cannot be confidently assumed; for example, when examining the effects of technological or socio-political changes (cf., Hoffmann et al., 2013; Verstraeten et al., 2016).

We implemented this latter approach by using a previously described method for the identification of the number and the location of joinpoints via Monte Carlo resampling (Kim et al., 2000), which is available via the R package *segmented* (Muggeo, 2003). We fitted models that included between zero and five joinpoints and selected the model with the number and location of joinpoints with the best fit to data on the basis of the Bayes information criterion. All models used an inverse variance weighted least squares regression, where the variance was derived from the estimated standard errors of the beta coefficients and the dispersion measures, respectively. The confidence intervals for the joinpoint locations were calculated using the Delta method, the standard choice for estimating the ratio of two random variables (Muggeo, 2003).

All calculations were performed using R (R Core Team, 2020). All datasets and code are available online (see Data and code availability).

## Results

We report our results in four parts. The first part illustrates the trends in life expectancy for the 23 Member States in the study during 1990–2017. The second part reports on the beta convergence analysis. The third part focuses on sigma convergence and the decomposition of dispersion. The final part examines regional mortality convergence in Czechia, Hungary, and Poland during 1992–2016.

### Trends in Life Expectancy in the Enlarged European Union

**Fig. 1:**
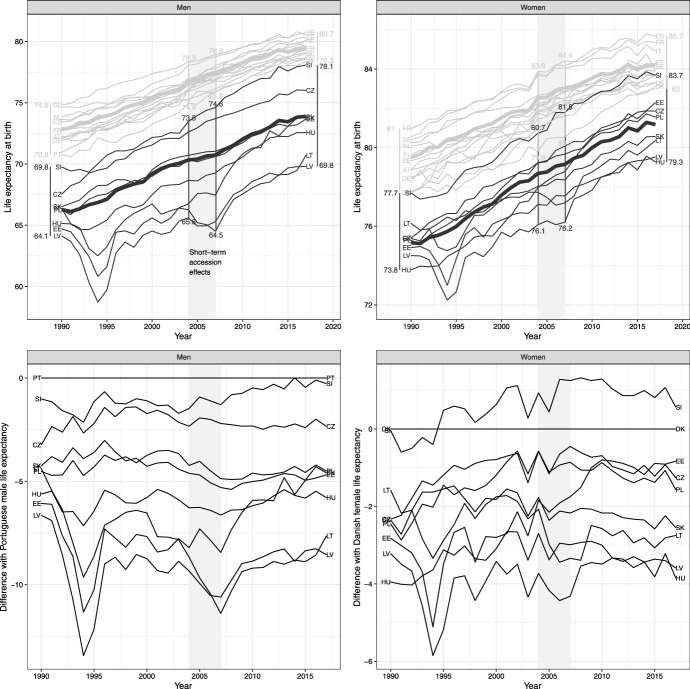
Trends in life expectancy in 23 EU Member States (top), and the difference (in years) between the life expectancy in the new Member States and the old Member State laggard—Portugal for men and Denmark for women (bottom), 1990–2017, by sex. The grey shaded area highlights the time window 2004–2007 associated with the short-term effects of the 2004 EU accession. The thick trend lines in the top figure illustrate the average life expectancy and the vertical lines its range in 1990, 2004, 2007, and 2017 for new and old Member States, respectively

The top half of Fig. 1 illustrates the trends in LE during the 1990–2017 period for the Member States included in our study. The average LE for NMS and OMS (thick lines in the top panel of Fig. 1) increased for both groups throughout 1990–2017. The gap between the two country groups became visibly smaller for women, while the trajectories for men run mostly parallel. The largely unchanged total range in LE between 1990 and 2017 for both sexes (vertical lines in the top panel of Fig. 1) also speaks against rapid overall mortality convergence. However, there is a noticeable increase in the LE range for NMS over the 1990–2017 period, particularly for men, where it nearly doubled, indicating mortality divergence between the NMS. For both sexes, the total LE range and the NMS LE range increased between 2004 and 2007, especially for men, due to the outlying performance of individual NMS countries.

Considering individual LE trajectories for NMS, we note that the NMS vanguard Slovenia caught up with the lagging OMS, Portugal for men and Denmark for women (see bottom half of Fig. 1). Latvia is the laggard for women for most of 1990–2017, and Latvia and Lithuania are laggards for men. Estonia exhibited the fastest improvement in LE for both sexes, particularly after the mid-2000s for women and after 2007 for men. Lithuanian female LE exhibits a dip that coincides with EU accession in 2004, as does the LE trajectory for Latvian men. No other NMS shows a clearly visible change in trend in 2004–2007.

### Beta Convergence in Life Expectancy

**Fig. 2:**
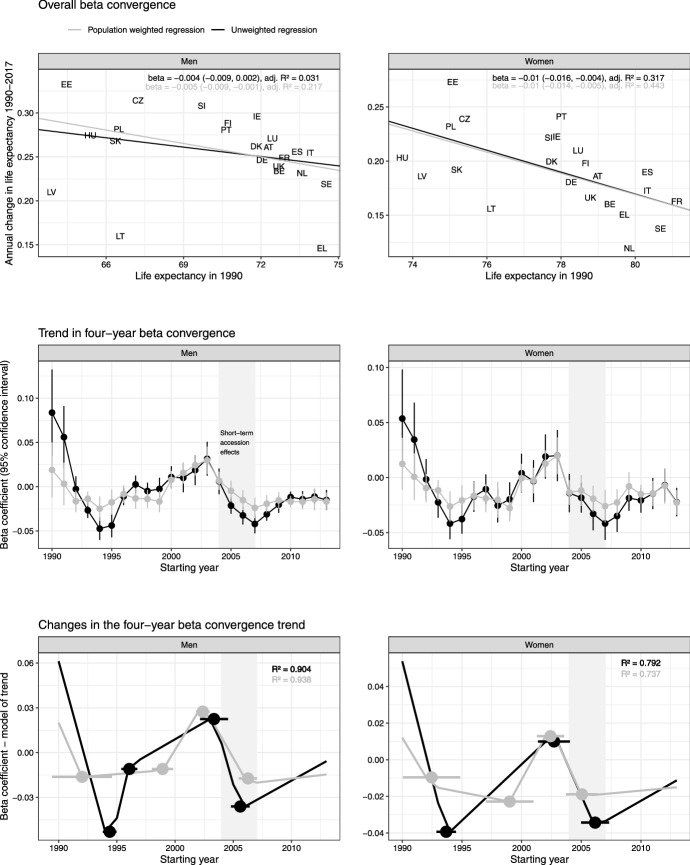
The association of LE in 1990 with its annual change during 1990–2017—overall beta convergence (top), the trend over time in the association between starting LE and the change in LE in the subsequent four-year period—four-year beta convergence coefficient (middle), and the joinpoint regression analysis estimating the year of the changes in the four-year beta convergence coefficient trend with the associated 95% confidence interval (bottom), by sex. The grey shaded area highlights the time window 2004–2007 associated with the short-term effects of the 2004 EU accession

The top row of Fig. 2 illustrates the association between life expectancy in 1990 and the annual change in life expectancy during 1990–2017. There is a significant inverse association between the two for women, which indicates that countries with initially lower LE saw the greatest improvements in LE (beta convergence). For men, the inverse association is significant only when considering the population-weighted regression. This is likely the consequence of the weighted regression reducing the influence of Latvia and Lithuania, both of which had a low initial male LE, but also a low growth in male LE.

The development over time of the association of LE with its change over the following four years is illustrated in the middle row of Fig. 2. While the weighted regression coefficients have a smoother trajectory (grey line) compared to the unweighted coefficients (black line), we can observe two distinct periods of beta convergence in both trajectories. The first took place during the 1990s, until it was interrupted by a period of divergence in the early 2000s. The second period of beta convergence began roughly at the time of accession in 2004 (women) or soon after (men). The precise years during which the beta coefficient was negative depend on the type of regression (weighted and unweighted) and sex, however, the overall pattern holds.

The bottom of Fig. 2 illustrates the results of the joinpoint regression, with which we examined whether any turning points in the above described trend fall within the time window 2004–2007. There was indeed a joinpoint located within this window for both sexes and both types of regression. However, only for the unweighted regression in men did the confidence interval of the joinpoint not cross outside the 2004–2007 window (joinpoint location = 2005.6, 95% CI [2004.8, 2006.4], *R*^2^ = 0.904). As the length of four-year intervals is arbitrary, we also performed a sensitivity analysis using six-year intervals (Supplementary Fig. 1). The sensitivity analysis confirms the results described above, however, all of the relevant joinpoint confidence intervals included time after 2007.

### Trends in Life Expectancy Dispersion in the Enlarged European Union

**Fig. 3:**
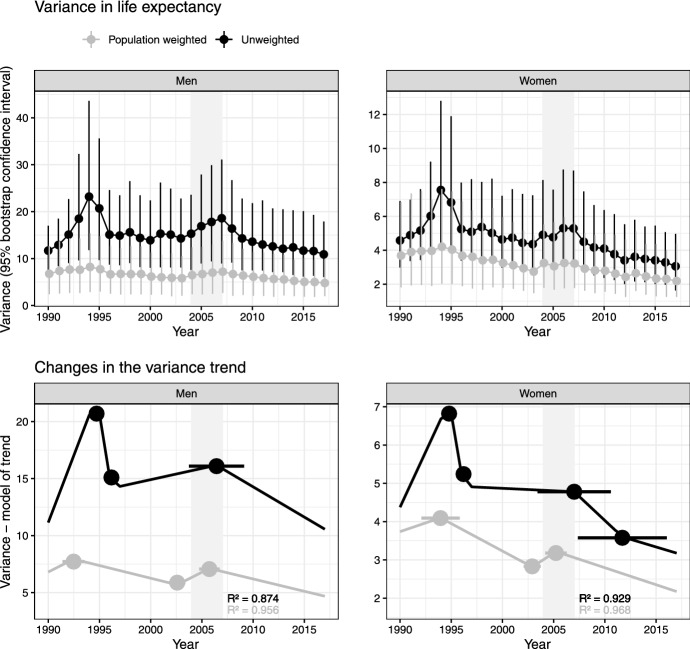
Variance in life expectancy, 1990–2017 (top) and the joinpoint regression analysis estimating the year of the changes in the variance trend with the associated 95% confidence interval (bottom), by sex. The grey shaded area highlights the time window 2004–2007, associated with the short-term effects of the 2004 EU accession

Figure 3 illustrates the trend in total dispersion in life expectancy as measured by unweighted variance (black line) and population-weighted variance (grey line). The dispersion followed an overall decreasing trend over time, more pronounced in women than in men. The overall trend was interspersed with peaks in the mid-1990s and late 2000s, which likely correspond to the pronounced fluctuations in LE in the less populous Baltic states in the mid-1990s and the mid-2000s. These interruptions also align with the changes in trend in beta convergence from the previous section. As expected, these trends are similar for both variance and the Theil index (Supplementary Fig. 2). The population-weighted versions of the measures are lower than their unweighted variants and their trajectories smoother, but otherwise exhibit the same trend as the unweighted versions, supporting the hypothesis that the observed peaks are driven mainly by the Baltic states.

The results of the statistical analysis of change in dispersion between 2017 and 1990 are reported in Supplementary table 2. The results show that the total dispersion in women significantly decreased for both unweighted and population-weighted measures (estimated decrease between 33.2% and 47.7%, depending on the measure), which indicates overall sigma convergence. For men, only population-weighted measures show a significant decrease in dispersion (estimated decrease of 29.1% in variance and 41.1% in the Theil index), which speaks for overall sigma convergence between the more populous countries only.

The bottom of Fig. 3 illustrates the results of the joinpoint regression for sigma convergence, with which we examined whether any turning points in the trend of variance occurred shortly after the 2004 EU accession (2004–2007). There were joinpoints located within this window for both sexes. However, only for the population-weighted variance for men did the confidence interval of the joinpoint not cross outside the 2004–2007 window (joinpoint location = 2005.7, 95% CI [2004.7, 2006.7], *R*^2^ = 0.956). A sensitivity analysis using the Theil index produced largely the same results, however, the confidence intervals for all joinpoints included time after 2007 (Supplementary Fig. 2). This means we cannot conclude that significant changes in sigma convergence trends occurred during or shortly after the 2004 EU enlargement.

The decomposition of variance (Table 1) shows that the between-country group component of dispersion dominated the total dispersion in both sexes throughout 1990–2017, and consequently that the reduction in the gap between NMS and OMS has been driving the overall sigma convergence. The within component on the other hand, which indicates differences between countries within OMS and NMS country groups, seems to have been responsible for the periodic peaks in dispersion observed on Fig. 3. The within component had tripled for men in NMS between 1990 and 2004 before settling in 2017 on a value double that of 1990. A similar pattern can be seen for the within component for women in NMS. In contrast, the within component in OMS was stable for women and had decreased over time for men.

**Table 1:** Decomposition of variance in life expectancy in 1990, 2004, and 2017

	Men			Women		
	1990	2004	2017	1990	2004	2017
Total variance, population-weighted, years squared	6.76	6.47	4.79	3.69	2.22	1.75
Between-country groups	5.98	5.48	3.99	2.64	2.01	1.18
New Member States	4.98	4.61	3.39	2.19	1.68	1.00
Old Member States	1.01	0.87	0.60	0.45	0.33	0.18
Within country groups	0.77	0.98	0.80	1.05	1.25	1.01
New Member States	0.16	0.49	0.33	0.09	0.16	0.15
Old Member States	0.62	0.49	0.47	0.96	1.09	0.86
Total variance, unweighted, years squared	11.4	15.1	10.7	4.49	4.84	3.05
Between-country groups	9.35	11.5	7.87	3.23	3.33	1.87
New Member States	6.10	7.47	5.14	2.10	2.17	1.22
Old Member States	3.25	3.98	2.74	1.12	1.16	0.65
Within country groups	2.01	3.64	2.85	1.27	1.51	1.18
New Member States	1.10	3.04	2.48	0.47	0.72	0.77
Old Member States	0.91	0.59	0.37	0.79	0.79	0.41

### Regional Convergence in Life Expectancy in Czechia, Hungary, and Poland

**Fig. 4:**
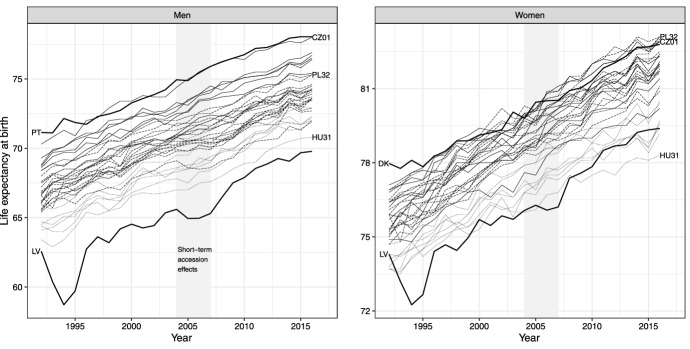
Trends in regional (NUTS 2) life expectancy in Czechia (solid grey lines), Hungary (dotted grey lines), and Poland (dashed grey lines), and national life expectancy in OMS laggards Portugal for men and Denmark for women and the NMS laggard Latvia (solid black lines), 1992–2016, by sex

Figure 4 illustrates regional LE trajectories for 8 NUTS 2 regions in Czechia (solid thin lines), 7 NUTS 2 regions in Hungary (dotted thin lines), and 31 NUTS 2 regions in Poland (dashed thin lines) for 1992–2016, by sex. For context, the plot also includes the national LE trajectories for Portugal and Denmark (laggard OMS for men and women, respectively) and Latvia (laggard NMS for both sexes). We can observe that the vanguard region in Czechia, the Prague region (CZ01), has almost caught up with the Danish LE for men, while the vanguard regions in Czechia (Prague region, CZ01) and Poland (Podkarpackie Voivodeship, PL32) have caught up and overtaken the female average LE in Denmark in the 2000s. On the other hand, the female LE in the laggard Hungarian region, Northern Hungary (HU31), has fallen behind Latvia since 2008.

None of the three countries saw a significant association between regional LE in 1992 and change in regional LE during 1992–2016, indicating no significant beta convergence between the regions for the three countries (Supplementary Fig. 3). Examining the trend in beta coefficients for four-year and six-year overlapping periods did not show notable fluctuation between periods of convergence and divergence for Czechia and Hungary shortly after the 2004 EU enlargement (see top half of Supplementary Fig. 4). However, our examination did reveal a change in trend for Polish regions, particularly for male LE, where the 2004 accession seemed to coincide with a transition to regional beta convergence. Nevertheless, the joinpoint regression shows that the relevant joinpoint confidence intervals extend outside the time window 2004–2007 for both four and six-year periods (four-year period joinpoint location: 2006.4, 95% CI [2005.3, 2007.6], *R*^2^ = 0.857; six-year period joinpoint location: 2006.1, 95% CI [2004.3, 2007.8], *R*^2^ = 0.908). We thus cannot conclude that significant changes in regional beta convergence in Czechia, Hungary, or Poland occurred during or shortly after the 2004 EU enlargement.

The analysis of regional sigma convergence for the three countries revealed large confidence intervals around the dispersion measures (Supplementary Fig. 4, bottom). The large confidence intervals associated with the estimated variance for each year are likely due to the large year-to-year variability in regional LE, which masks any potential underlying trends. This makes detecting significant changes in the trend shortly after the 2004 EU enlargement using joinpoint regression highly unlikely.

## Discussion

### Summary of Main Results

We examined mortality convergence in the post-2004 EU during 1990–2017, with a particular focus on the possible short-term effects of the eastward 2004 enlargement during 2004–2007. We found no compelling evidence that EU accession influenced the process of mortality convergence between new and old Member States, or within the three studied new Member States (Czechia, Hungary, and Poland) over the short term. While there was overall beta and sigma convergence in women and men when considering population-weighted measures at the national level during 1990–2017, our analysis of trends in both sigma and beta mortality convergence measures resulted in insufficient statistical evidence to claim these trends were significantly changed at the time of EU accession or soon after. We found no overall regional mortality convergence in Czechia, Hungary, and Poland during 1992–2016, nor did the measures of mortality convergence significantly change in 2004–2007.

### Interpretation of Results

Our finding that the 2004 EU accession did not significantly affect the rate of mortality convergence may be explained by three factors. First, the process of the 2004 EU accession unfolded over a decade and many of the associated changes to the socio-economic context were already in effect prior to the moment of accession. Second, the 2004 accession process largely failed to harmonise social and health policy between the new and old Member States. Third, the accession-related mortality effects, if they exist, likely differed over time and between countries and regions, which makes them difficult to detect at the level of the enlarged EU as a whole.

Related to the first factor, a detailed look at the 2004 EU accession process reveals that major socio-economic changes in accession countries took place during the pre-accession phase already. That is, all of the NMS in our study signed the so-called “Europe Agreements”, which entered into force in 1994 (Hungary and Poland), 1995 (Slovakia and Czechia), 1998 (the Baltic States), and in 1999 (Slovenia). These agreements removed most barriers to free trade, liberalised the movement of services and capital, and allowed limited participation of accession countries in EU programmes (cf., Ministry of Foreign Affairs of the Republic of Estonia 2009). Furthermore, the European Commission’s evaluation of the progress of the accession countries, the *Agenda 2000* report (European Commission, 1997), highlighted that Czechia, Estonia, Hungary, Poland, and Slovenia had already made significant progress in meeting the Copenhagen criteria by 1997. This indicates that substantive policy changes had already occurred in these accession countries by that time. Additionally, previous research on foreign direct investment in the accession countries, which is a key mechanism of economic integration, also found an anticipatory effect of EU membership: namely, that investment in these countries increased soon after the announcement of the start of the accession process in 1993 (Bandelj, 2010; Bevan & Estrin, 2004; Clausing & Dorobantu, 2005). Thus, both the policy changes and increased investment that are typically associated with faster LE growth and mortality convergence were already partly in place in the accession countries by the time they became official members of the bloc in 2004.

Related to the second factor, EU accession did not lead to a rapid harmonisation of social and health policy between the new and old Member States. Before and during the 2004 EU accession, new Member States adopted different socio-economic institutions (Zweynert & Goldschmidt, 2006), including policies regarding health, education, and social welfare. This is the result of health and social policies being explicitly excluded from the harmonisation of laws and regulations in the Member States by the Treaty of Nice (Oksanen, 2013), thus making them largely Member State decisions and exempt from the harmonising pressures of EU accession. A key example from our study are the Baltic states, where differences in healthcare reforms and health policies contributed to divergent mortality trends in these countries during 2000–2007, despite the participation of all three countries in EU accession procedures at that time (Jasilionis et al., 2011).

Related to the third factor, national and regional characteristics other than domestic policy may also shape how European integration influences mortality conditions, resulting in a complex pattern of effect that may be difficult to detect at the macro level. For example, western Polish regions attracted more foreign investment before 2004 due to their geographic location (Cieślik, 2005), while the more disadvantaged eastern Polish regions benefitted from the cohesion policy transfers that became available after 2004 (Cieślik et al., 2010). While both of these mechanisms are related to European integration, they acted in different regions at different times, and therefore did not visibly influence mortality during a narrow time window. Other national and regional characteristics—like urbanicity and rurality (Allan et al., 2019), net outmigration (Tunstall et al., 2016), and the level of unemployment (Laliotis & Stavropoulou, 2018) – have also been shown to be associated with population health outcomes. Their interactions with European integration require further study, preferably in the form of in-depth case studies of single regions or countries that may uncover complex and context-specific networks of cause and effect.

### Limitations

Our study has a number of limitations. The first is the missing regional life expectancy data, which prevented us from incorporating the subnational experience more broadly. Because sharing data with Eurostat was voluntary between 1990 and 2012 (Eurostat 2020b), compiling and sharing these data may have not been a high priority for some Member States. Another common reason for missing data is that the borders of administrative units changed frequently.

The second limitation is the influence of increased migration following the EU accession on the accuracy of the mortality rate estimates, and, hence, the estimated LE. Although migration from the NMS to the central European OMS (e.g. Hungary to Austria) was limited during 2004–2006 by transitional measures for the free movement of workers (European Commission, 2006), intra-country migration and migration to other OMS (e.g. Poland to the UK) was widespread, and may have contributed to the misestimation of mortality rates due to the overestimation of the population in the net outmigration countries and regions.

The third limitation is the difficulty we faced in interpreting the trends in mortality convergence due to confounding by other events, including the Baltic mortality crisis (1992–1997) (Jasilionis et al., 2011), and the Great Recession and the European sovereign debt crisis (2007 and after). We attempted to mitigate the impact of the first by including the population-weighted dispersion measures, which have been shown to be less sensitive to the experiences of outliers with small population sizes. We attempted to mitigate the second by focusing on the short-term direct effects of EU membership (i.e. those that could have changed LE trends during the 2004–2007 period). This may, however, have resulted in excluding EU accession mechanisms that act with longer delays and over diffuse areas, and thus an underestimation of convergence.

The fourth point is that our analysis does not account for trends in all mortality determinants. While changes in economic development, health and social policy, health-related behaviours and other factors certainly influence mortality conditions independent of EU accession or act as mediators in the countries and regions analysed, it was not our objective to fully explain mortality or mortality convergence trends. This would have been unfeasible due to limited data on mortality determinants. Instead, we focused on identifying short-term fluctuations in mortality convergence measures at and around the time of accession.

The final limitation is related to the use of joinpoint regression in small sample sizes. The joinpoint regression is strongest when data on many units over a long period of time are available. In contrast, our sample was small both in terms of the number of countries and regions included, as well as the length of time considered. However, by combining joinpoint regression with other statistical approaches that are useful in small samples, such as resampling as we did, we were able to reliably detect rapid shifts in mortality convergence that occured during the Baltic mortality crisis (see Figs. 2 and 3). Therefore, we are reasonably certain not to have missed a similarly rapid and consequential change in mortality convergence associated with short-term consequences of EU accession.

### Future Directions for Research and Policy Recommendations

Our work identified several interesting future research directions. First, it may be useful to examine potential EU membership effects in a series of regional case studies. This approach would allow the researchers to cope with the heterogeneity in the extent and the onset of EU membership effects, and it could offer insights into how national institutions and policies and regional geographic, demographic, and economic characteristics shape the effects of European integration on LE trajectories. Second, while specific components of European integration may have positively influenced mortality convergence, their effects were obscured by our examination of the effects of European integration in general. It may be interesting to closely examine the effects of particular aspects of European integration and EU membership on mortality convergence, particularly the creation of new economic networks and regional financial flows (e.g. cross-border investment and ownership of regionally important firms; or the regional receipt of cohesion funds).

Based on our results, we offer two recommendations to policy-makers interested in supporting mortality convergence in the enlarged EU. First, policy areas that are likely to play important roles in population health, and that were previously part of the “soft” *aquis* (including the European social model), should become part of the “hard” *acquis* in order to reduce undue cross-country variability in labour conditions that may be undermining the health benefits of economic integration by introducing uncompensated employment shocks (Bíró & Branyiczki, 2020). Second, future accession procedures may benefit from regionally tailored approaches to financial and technical assistance, which would consider the balance of benefits and harms of greater political and economic integration, and offer additional support (e.g. sufficient unemployment and retraining support) for regions that may be harmed by the process. These two recommendations should help to maintain the overall benefits of European integration, while also maximising the distribution of health gains that can help prevent the emergence of left-behind regions. Finally, we recommend that policy-makers support emerging efforts to improve the infrastructure for mortality research at the regional level, since available and accessible data on regional health outcomes, particularly causes of death and health determinants, are currently lacking. An expansion of the reporting cycles (e.g. State of Health in the EU (OECD/EU 2018)) with information on geographic disparities in health at the subnational level could speed up this process.

## Conclusion

Our study examined the short-term effects of European integration—and EU membership in particular—on mortality convergence, focusing on the case of the 2004 enlargement. We found that the accession in 2004 did not visibly impact the overall process of mortality convergence over the short term, possibly because of the more important influence of country and region-specific policies and characteristics. The interaction of geographic, demographic, and economic characteristics of new Member States and regions with the mechanisms of European integration require further study, preferably in the form of in-depth case studies that may uncover context-specific networks of cause and effect. Based on our findings, we recommend that policy-makers consider including key social policies as part of the standard *acquis communautaire*, provide tailored support to regions in accession countries, and support efforts to improve the data infrastructure in the EU at the subnational level.

## Supplementary Information

Below is the link to the electronic supplementary material.

Supplementary file1 (PDF 1427 kb)

## Data Availability

All code and data associated with this paper is available online at https://github.com/rhrzic/EUJP_EUMortalityConvergence
