# Supplementary material for: The Short-Term Effects of European Integration on Mortality Convergence: A Case Study of European Union’s 2004 Enlargement
Source: Eur J Popul. 2021 Oct 7;37(4-5):909–31. doi: 10.1007/s10680-021-09596-y (PMC8575723; doi:10.1007/s10680-021-09596-y)
Supplement: Supplementary file 1 — Supplementary file1 (PDF 1427 kb) [file 10680_2021_9596_MOESM1_ESM.pdf]

The short-term effects of European integration on mortality convergence: a case study of European Union's 2004 enlargement

Supplementary material

Contents

Supplementary Table 1. Regional data availability for 1990-2016.

Supplementary Figure 1. The trend over time in the association between starting LE and the change in LE in the subsequent six-year period – six-year beta convergence coefficient (top), and joinpoint regression analysis estimating the year of the changes in the trend in the six-year beta coefficient with the associated 95% confidence interval (bottom), by sex.

Supplementary Table 2. Bootstrap confidence intervals of change in dispersion measures during 1990-2017.

Supplementary Figure 2. Theil index of life expectancy, 1990-2017 (top) and joinpoint regression analysis estimating the year of the changes in the Theil index trend with the associated 95% confidence interval (bottom), by sex.

Supplementary Figure 3. The association between regional LE in 1992 and its annual change during 1992-2016, by sex and country.

Supplementary Figure 4. The trend over time in the association between starting regional LE and the annual change in regional LE in the subsequent four-year period in Czechia, Hungary, and Poland, by sex (top). Trend over time in variance in regional LE, by country and sex (bottom).

Supplementary Table 1. Regional data availability for 1990-2016.

Country	*Number of NUTS 2 regions, 2016 classification (regions with interruptions > 1 year)*	*Comments*
Austria	9 (0)	1995 missing for all regions.
Belgium	11 (0)	
Czechia	8 (8)	No data available 1990-1991. Complete after 1992.
Germany	38 (38)	No data available 1990-2001.
Denmark	5 (5)	No data available 1990-2006.
Estonia	1 (0)	
Finland	5 (0)	
France	27 (5)	Data mostly missing for the five overseas regions.
Greece	13 (0)	Data missing for nine regions in 1990. Complete after 1991.
Hungary	8 (2)	HU10 (Budapest region) has been deprecated and split into HU11 and HU12 in the 2016 NUTS classification, but data in this configuration is missing for 1990-2013. We therefore used the older classification, which includes 7 regions.
Ireland	3 (3)	No data available 1990-2012.
Italy	21 (2)	ITI3 (Marche) and ITH5 (Emilia-Romagna) were reformed in 2010, their 1990-2000 data is unavailable.
Lithuania	2 (2)	No data available 1990-2001.
Luxembourg	1 (0)	
Latvia	1 (1)	No data available 1990-2001.
Netherlands	12 (12)	No data available 1990-2000
Poland	17 (7)	No data available for 2001, which was addressed by interpolation. Seven new regions were introduced in the 2016 classification; data for them 1990-2013 is missing.
Portugal	7 (0)	

Spain	19 (0)	
Sweden	8 (0)	
Slovenia	2 (2)	No data available 1990-2007.
Slovakia	4 (4)	No data available 1990-1996.
UK	41 (41)	No data available 1995-1998. UKD6 & UKD7 also miss 1993-1994 & 1999-2001 (so in total all data 1990-2001). Same goes for London (UKI3-7), where all data 1990-2012 is missing. For the old Scottish regions (UKM2,3,5,6) 1993-1994, 1999, and 2017 is missing.

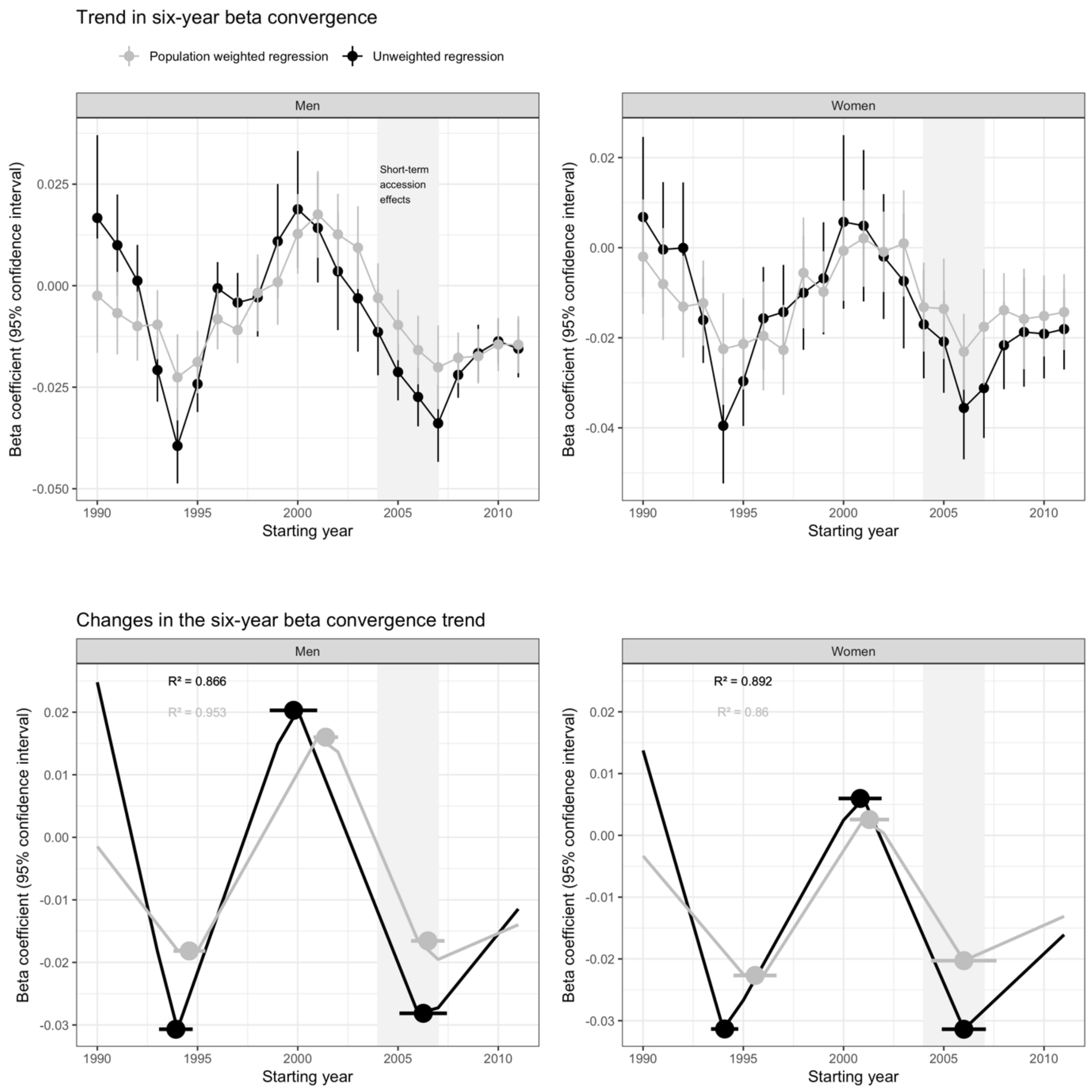

Supplementary Figure 1. The trend over time in the association between starting LE and the change in LE in the subsequent six-year period – six-year beta convergence coefficient (top), and joinpoint regression analysis estimating the year of the changes in the trend in the six-year beta coefficient with the associated 95% confidence interval (bottom), by sex. The grey shaded area highlights the time window 2004-2007, associated with the short-term effects of the 2004 EU accession.

Supplementary Table 2. Bootstrap confidence intervals of change in dispersion measures during 1990-2017

		Difference 2017-1990 (95% bootstrap CI)			
		Variance	*Theil index*	*Variance*	*Theil index*
		Unweighted		*Population weighted*	
Relative difference (%)	Men	-6.99 (-36.7, 38.90)	-22.5 (-49.1, 14.6)	-29.1* (-44.1, -14.6)	-41.1* (-53.3, -28.9)
	Women	-33.2* (-59.9, -8.85)	-41.1* (-63.5, -18.4)	-40.7* (-59.3, -16.0)	-47.7* (-64.9, -26.9)
Absolute difference	Men	-0.816 (-4.08, 3.780)	-0.000255 (-0.000599, 0.000064)	-1.97* (-3.78, -0.588)	-0.000275* (-0.000513, -0.00008)
	Women	-1.52* (-2.91, -0.555)	-0.000149* (-0.000264, -0.000069)	-1.50* (-3.15, -0.436)	-0.000143* (-0.000288, -0.000047)

*Difference is significantly different from 0.

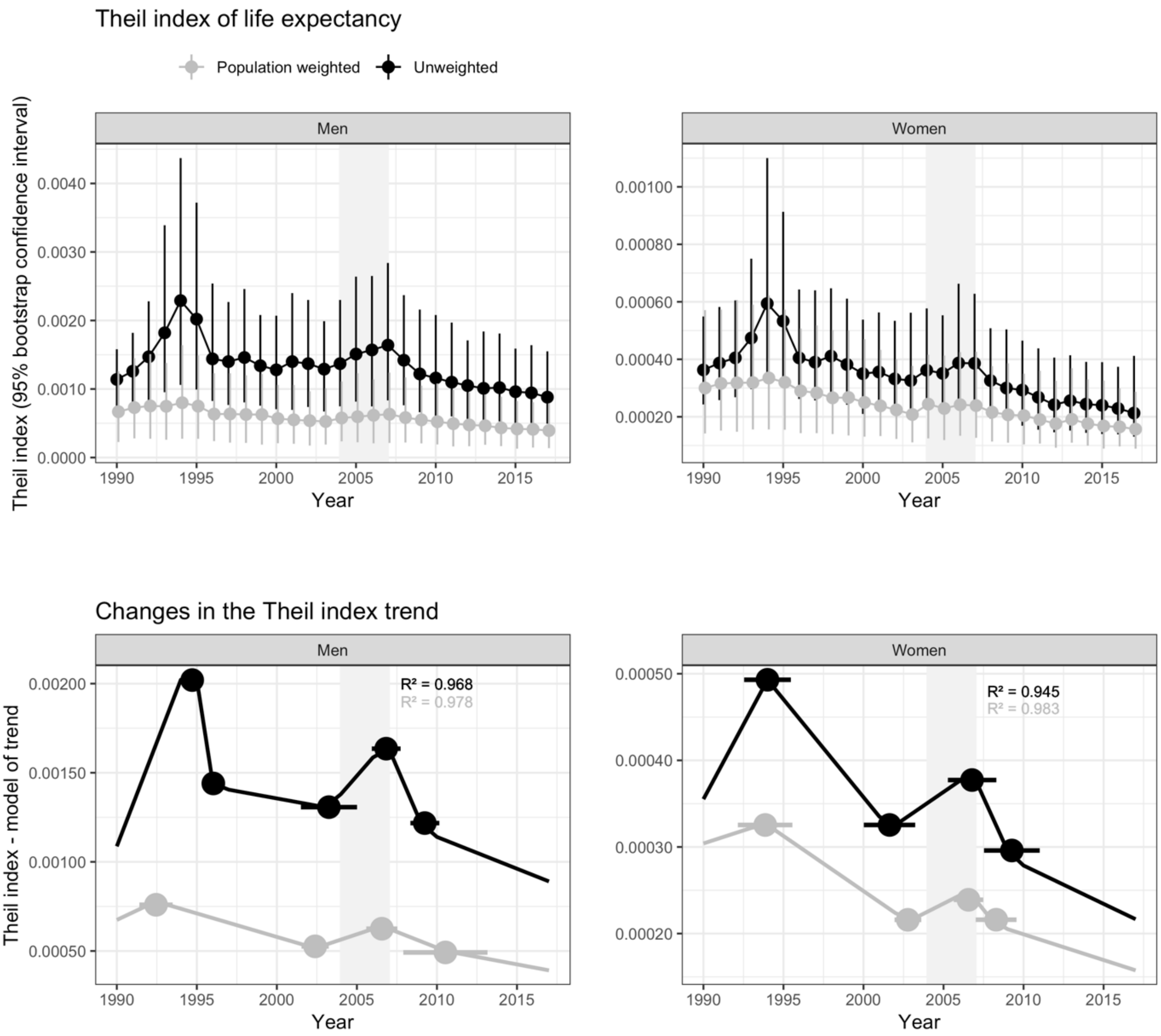

Supplementary Figure 2. Theil index of life expectancy, 1990-2017 (top) and the joinpoint regression analysis estimating the year of the changes in trend in the Theil index with the associated 95% confidence interval (bottom), by sex. The grey shaded area highlights the time window 2004-2007, associated with the short-term effects of the 2004 EU accession.

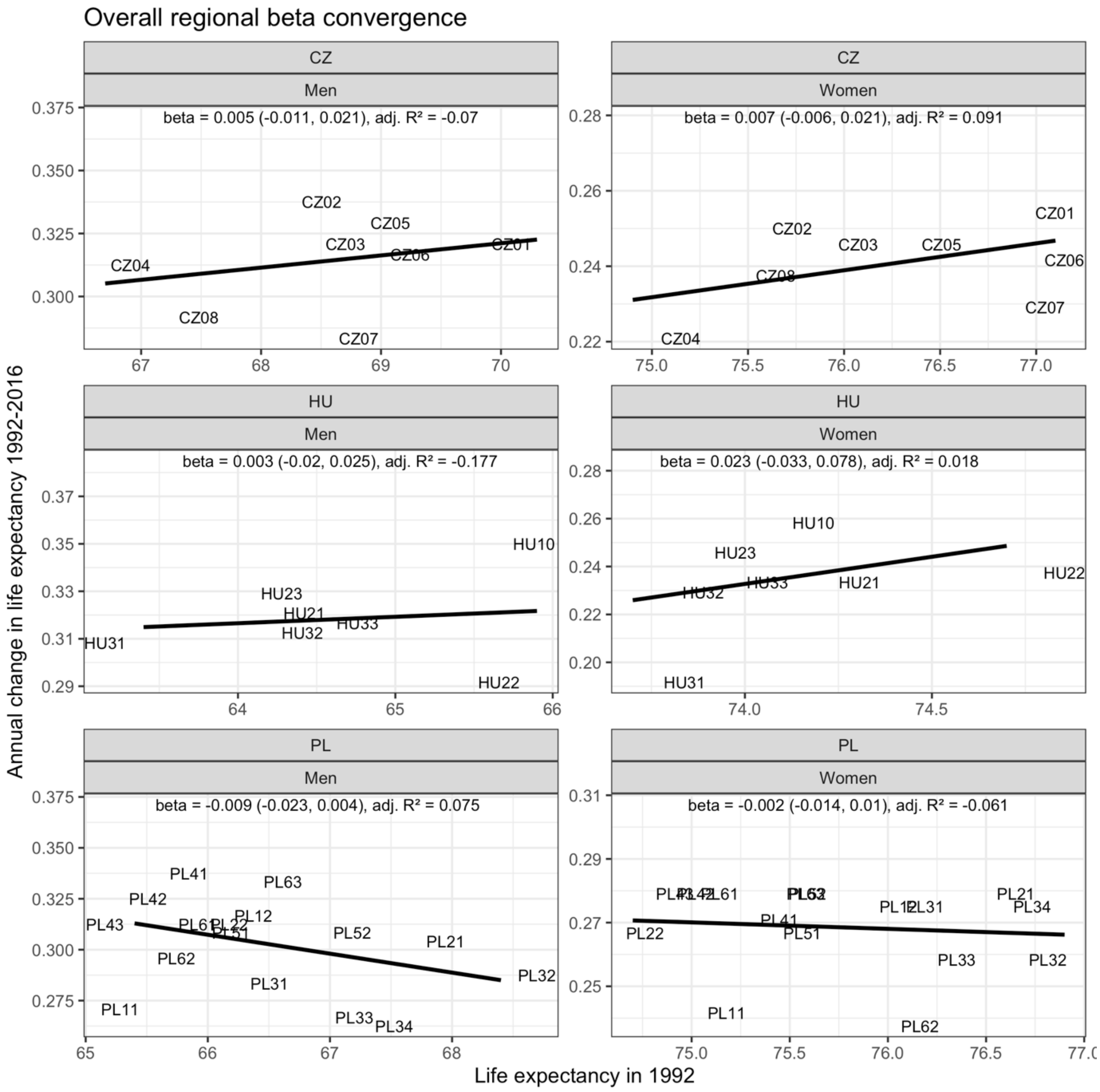

Supplementary Figure 3. The association of regional life expectancy in 1992 and its annual change in Czechia, Hungary, and Poland during 1992-2016, by sex. All plots are annotated with the beta coefficient estimate, the associated 95% confidence interval, and adjusted R^2.

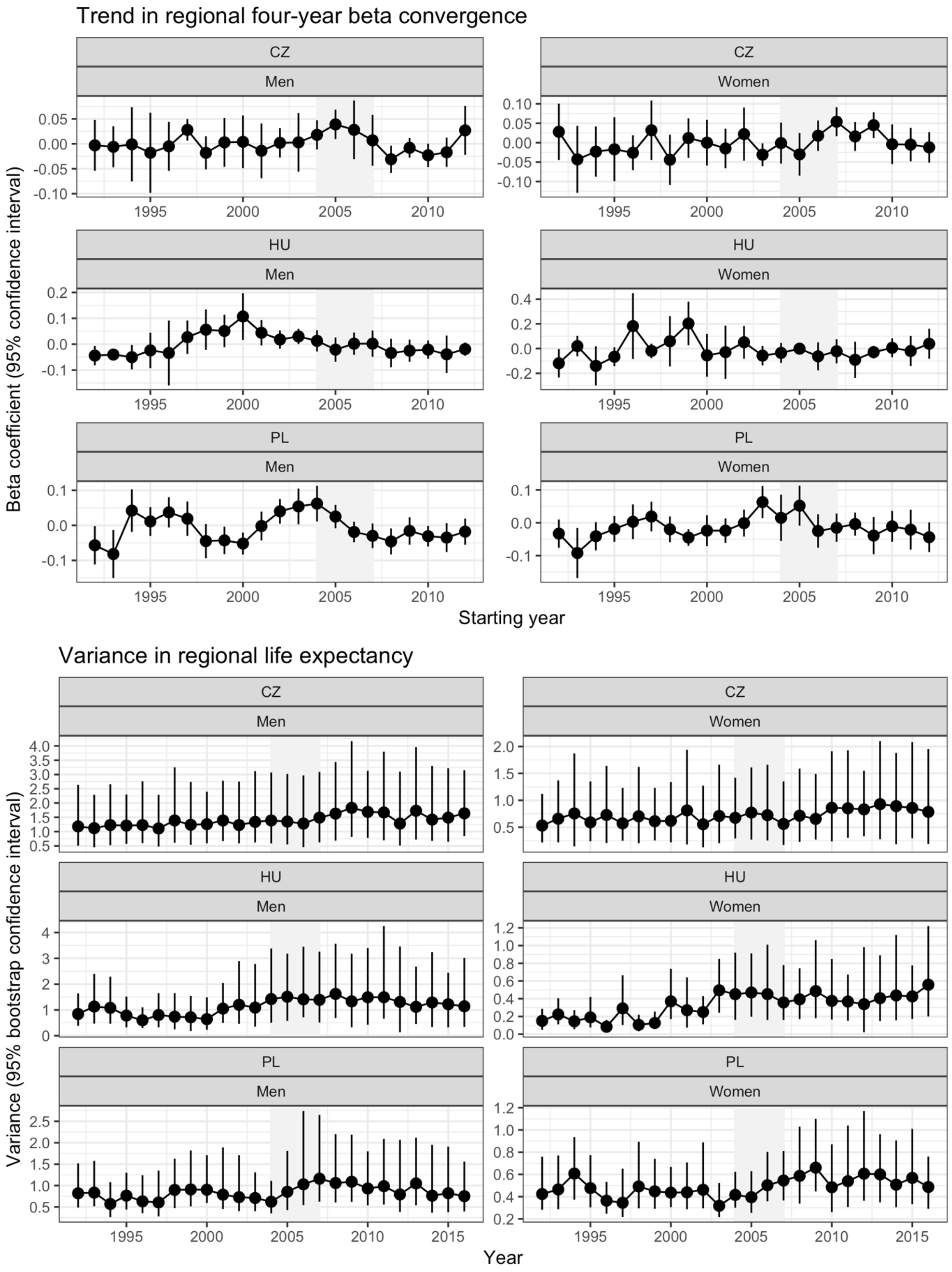

Supplementary Figure 4. The trend over time in the association between starting regional LE and the annual change in regional LE in the subsequent four-year period in Czechia, Hungary, and Poland, by sex (top). Trend over time in variance in regional LE, by country and sex (bottom).
